# Cure by Obliteration

**Published:** 1912-04-06

**Authors:** 


### CURE BY OBLITERATION.

To give functional rest to a diseased part is a classic and obvious—the attributes are too often conjoined—principle of treatment whose application modern surgery has been able to extend far beyond any limits Hilton can have imagined. The dire progress of advanced cancer of the lower bowel may, as is well known, be checked, palliated, by a well-executed colotomy. Lately we have heard a good deal of a new treatment of consumption which, unlike almost all innovations under that head, really seems to have something in it, although its scope must be limited to relatively few cases. The injection with appropriate technique of nitrogen gas into one pleural cavity, by throwing a diseased lung out of use, may stay also the activity of the disease there. By repeating this process at intervals so as to keep up its effect, and then gradually allowing function to be regained, this quiescence of the pulmonary tuberculous process may even be brought to deepen into obsolescence: in short, permanent arrest may ensue, and this, if reports be correct, in cases refractory to the usual methods of treatment. There is, however, another surgical intervention, also, as it happens, in connection with tuberculous disease, of which—so we learn from the current number of the *Internationales Centralblatt für die gesammte Tuberkuloseforschung*—the first case was reported as lately as last year. Willems gave an account to the Belgian Royal Academy of Medicine of a man who for five years had suffered from that very grave affection, tuberculosis of the bladder. At the operation the right kidney was removed, being found hopelessly diseased. The ureter of the left one was then ligatured and divided and the organ itself applied to the lumbar region and a urinary fistula made. By the next day all the distressing bladder symptoms had disappeared, and in a few months' time the patient had gained twenty kilograms in weight, the fistula causing no great inconvenience except for one or two unimportant abscesses. The sequel of the case is not so happy, for fifteen months after the operation the man, who had celebrated the new year by getting very drunk, died by the end of January with uræmic symptoms, and at the post mortem the kidney was found in a suppurative condition. The tuberculous ulceration of the bladder, formerly very severe, was however quite healed, and the bladder itself resembled a small, atrophied uterus. Accounts of further experience of this operation would be very interesting. At present, in spite of tuberculin and other none-too-effectual specifics, we read of the rapid disappearance of broken-down vesical tubercle with something of the same feelings with which, in the pre-Listerian era, our predecessors must have looked on the rare healing by first intention of a penetrating wound of a knee-joint. It is an old story now that, thanks to the great man who lately, as Meredith said of Garibaldi, returned to the earth that gave him, surgeons are being enabled more and more to mend the human frame as mechanical engineers mend their apparatus. Although the range of expedient in the one case can never be so uncontrolled as in the other, yet, seeing the superior complexity of nature to that of art, it must inevitably soon come to be a great deal wider. To descend to a rather lower plane of reflection, the prestige of such power is likely to be highly useful to the medical profession in the present (and future) rebellious times.

